# Optimal Timing of Radiotherapy Following Gross Total or Subtotal Resection of Glioblastoma: A Real-World Assessment using the National Cancer Database

**DOI:** 10.1038/s41598-020-61701-z

**Published:** 2020-03-18

**Authors:** Samantha M. Buszek, Karine A. Al Feghali, Hesham Elhalawani, Neil Chevli, Pamela K. Allen, Caroline Chung

**Affiliations:** Department of Radiation Oncology, University of Texas MD Anderson Cancer Center, Houston, Texas United States

**Keywords:** Outcomes research, CNS cancer

## Abstract

Treatment for glioblastoma (GBM) includes surgical resection and adjuvant radiotherapy (RT) and chemotherapy. The optimal time interval between surgery and RT remains unclear. The National Cancer Database (NCDB) was queried for patients with GBM. Overall survival (OS) was estimated using Kaplan-Meier and log-rank tests. Univariate (UVA) and multivariable Cox regression (MVA) modeling was used to determine predictors of OS. A total of 45,942 patients were included. On MVA: younger age, female gender, black ethnicity, higher KPS, obtaining a gross total resection (GTR), MGMT promoter-methylated gene status, unifocal disease, higher RT dose, and RT delay of 4–8 weeks had improved OS. Patients who underwent a subtotal resection (STR) had worsened survival with RT delay ≤4 weeks and patients with GTR had worsened survival when RT was delayed >8 weeks. This analysis suggests that an interval of 4–8 weeks between resection and RT results in better survival. Delays >8 weeks in patients with a GTR and delays <4 weeks in patients with a STR/biopsy resulted in worse survival. This impact of time delay from surgery to RT, in conjunction with extent of resection, should be considered in the clinical management of patients and future designs of clinical trials.

## Introduction

Glioblastoma (GBM) is the most common primary malignant brain tumor in adults. Current standard of care treatment for patients 70 years or younger and with good performance status, per the National Comprehensive Cancer Network (NCCN), includes maximal safe surgical resection with image-verified complete resection, followed by adjuvant radiotherapy (RT) and chemotherapy^1^. Several prior studies have demonstrated an improved progression-free survival with more complete surgical resections^2–6^. Under the same assumption that maximum cytoreductive treatment provides benefit to patients and, given the aggressive and rapidly progressive nature of this disease, many clinicians seek to minimize the time delay between surgery and initiation of RT. However, in the modern era, there are potentially numerous factors that could delay time to initiation of RT, including: evolution of practice that incorporates molecular/genetic testing and consideration of enrollment to clinical trials that requires additional testing and even central pathology review. This study provides information about the possible clinical impact of time delays for these various reasons between surgical resection and the start of adjuvant radiation therapy for patients with GBM.

The optimal time interval between surgery and the initiation of adjuvant therapy; however, remains unclear. Cancer in several non-central nervous system (CNS) sites, including head and neck cancer and breast cancer, have increased local-regional recurrence rates when adjuvant RT is delayed^7^. To our knowledge, in patients with GBM, two retrospective series have indicated that a delay in initiating adjuvant therapy worsened survival^8,9^; nine series, including a SEER analysis, indicated that a delay had no significant impact on outcomes^10–18^; and eleven studies reported that a delay of varying time amounts provided a survival advantage^19–29^. These conflicting conclusions may be due to the retrospective nature of the majority of these studies, small sample sizes, outdated therapies, and the differing time points chosen for data analysis to define a delay.

Therefore, this study aims to use a large cohort from the National Cancer Database (NCDB) to identify predictors for and clinical impact of time from surgical resection to initiation of RT in patients with newly diagnosed GBM.

## Materials and Methods

### Population

The National Cancer Database (NCDB) is a joint project of the Commission on Cancer (CoC) of the American College of Surgeons and the American Cancer Society. It is a hospital-based registry that captures approximately 70% of newly-diagnosed cancer cases in the United States and Puerto Rico and draws data from >1500 commission-accredited cancer programs. This program originated in 1989 and now contains approximately 34 million records. Data registries contain patient characteristics, cancer staging and tumor histological characteristics, type of first course treatment administered, and outcomes. The American College of Surgeons and the CoC have not verified and are not responsible for the analytic or statistical methodology used, or for the conclusions drawn from these data by the investigator. This NCDB analysis was approved by the institutional review board at MD Anderson Cancer Center and all methods were performed in accordance with the relevant guidelines and regulations. Additionally, a waiver of informed consent was obtained as the information in the Commission on Cancer’s NCDB is de-identified.

**Figure 1:**
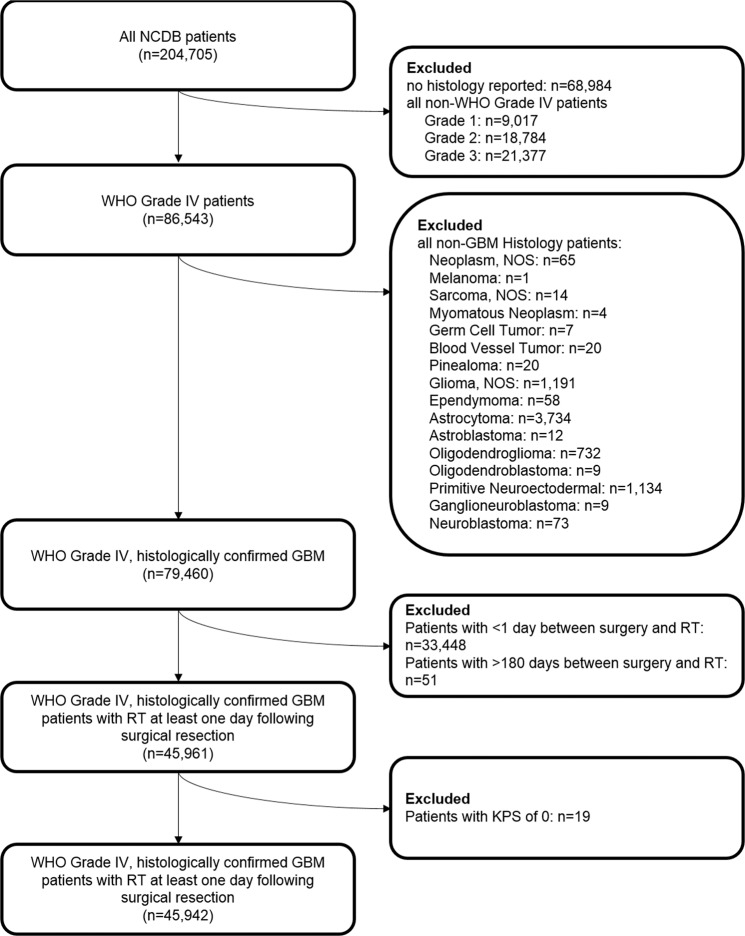
Cohort derivation. NCDB = National Cancer Database; WHO = World Health Organization; GBM = Glioblastoma; NOS = not otherwise specified; RT = radiotherapy; KPS = Karnofsky Performance Status.

We queried the NCDB User File for adult patients with primary glioblastoma treated between 2004–2015 using the International Classification of Diseases for Oncology histology codes 9440 and site-specific codes C720-3. Summary of cohort selection is detailed in Fig. 1. We only included patients with World Health Organization (WHO) Grade IV disease, and histologic confirmation of glioblastoma, NOS, giant cell glioblastoma, and gliofibroma. We excluded patients with gliosarcoma or choroid glioma, as well as other non-GBM histologies. We calculated the time between surgical resection and initiation of radiation as the subtraction between the variables: ‘Time from diagnosis to radiation’ and ‘Time from diagnosis to surgical resection’. We excluded all negative and zero day time points to ensure that radiotherapy was performed following surgery, as well as time intervals greater than 6 months as these patients were likely treated with radiotherapy for progressive disease. Time interval between surgery and the start of RT were grouped into ≤4 weeks, 4.1–6 weeks, 6.1–8 weeks, and >8 weeks. These time intervals were selected for their prevalence in the published literature, and for their ease of clinical application. Resection status analysis was performed on patients with a reported gross total resection (GTR) or subtotal resection (STR)/biopsy, only.

### Key covariates

Covariates incorporated into our analysis were selected *a priori* and included age, KPS, gender, ethnicity, surgical resection type, MGMT promoter-methylation status, unifocal/multifocal, total radiation dose, survival status, and follow-up time.

### Statistical analyses

Statistical analyses were performed using Stata/MP statistical software (version 15.1; Stata, College Station, TX). Univariate (UVA) and multivariable (MVA) modeling with Cox regression analysis was used to determine predictors of overall survival. Statistically significant predictor variables on UVA were included in MVA models. Overall survival was estimated using Kaplan-Meier and log-rank test methods. Statistical tests were based on a 2-sided significance level, and a P value ≤ 0.05 was considered to be statistically significant.

## Results

### Population characteristics

A total of 45,942 patients met inclusion criteria and were included in the analysis. The mean age at diagnosis was 61 years (range 18–90 years). The majority of patients were male (59%), of white ethnicity (91%), and had an unknown Karnofsky Performance Status (KPS; 55%). Only 7% of patients had a reported KPS > 70 in this patient cohort.

### Tumor and treatment characteristics

A total of 11,470 patients underwent a gross total resection (GTR) and 13,594 underwent a subtotal resection or biopsy (STR). The median time interval from resection to RT was 29 days (range 1–179 days). Almost half of the cohort (47%) were initiated on RT at <4 weeks from surgical resection, 38% had a delay of 4.1–6 weeks from surgery, 10% had a delay of 6.1–8 weeks, and 5% had a delay of over 8 weeks from resection before beginning RT. Cohort characteristics by RT delay groupings are summarized in Table 1.

**Table 1:** Descriptive statistics and associations between radiation delay groups.

n (%)	RT Delay following Surgical Resection Groupings					p-value
	All Patients (n = 45,942)	≤4 weeks (n = 21,804)	4.1–6 weeks (n = 17,294)	6.1–8 weeks (n = 4,471)	>8 weeks (n = 2,373)	
Age, median (range), years	61 (18–90)	62 (18–90)	61 (18–90)	62 (18–90)	60 (18–90)	<0.001
KPS						0.001
≥70	3,304 (7)	1,434 (7)	1,422 (8)	307 (7)	141 (6)	
<70	615 (1)	267 (1)	245 (1)	72 (2)	31 (1)	
Gender, male	26,974 (59)	12,906 (59)	10,077 (58)	2,632 (59)	1,359 (57)	0.136
Ethnicity, white	41,899 (91)	20,116 (92)	15,798 (91)	3,925 (88)	2,060 (87)	<0.001
Surgical Resection						<0.001
GTR	11,470 (25)	4,622 (21)	4,880 (28)	1,325 (30)	643 (27)	
STR/biopsy	13,594 (30)	6,077 (28)	5,444 (31)	1,402 (31)	671 (28)	
No Surgery	231 (0)	110 (1)	80 (1)	26 (1)	15 (1)	
Surgery NOS/Unknown*	20,647 (45)	10,995 (50)	6,890 (40)	1,718 (38)	1,044 (44)	
Tumor Focality						<0.001
Unifocal	20,511 (45)	8,510 (39)	8,605 (50)	2,280 (51)	1,118 (47)	
Multifocal	3,859 (8)	1,927 (9)	1,421 (8)	359 (8)	152 (6)	
Unknown	21,572 (47)	11,367 (52)	7,270 (42)	1,832 (41)	1,103 (47)	
MGMT promoter-Methylated						<0.001
Yes	2,743 (6)	1,137 (5)	1,181 (7)	297 (7)	128 (5)	
No	4,132 (9)	1,633 (8)	1,861 (11)	448 (10)	190 (8)	
Unknown	39,067 (85)	19,034 (87)	14,252 (82)	3,726 (83)	2,055 (87)	
RT Dose, median (range), Gy	60 (20–75)	60 (20–75)	60 (20–75)	60 (20–75)	60 (20–75)	<0.001

RT: radiotherapy; RT Delay: time from surgical resection to initiation of RT; KPS: Karnofsky Performance Status; GTR: Gross Total Resection; STR: Subtotal Resection; Gy: Gray; NOS: not otherwise specified.

*Patients treated with surgical resection of their glioblastoma; however, resection extent was not reported. These patients were included in the primary analysis evaluating the time interval between surgery and RT, but they were not included in the secondary analysis evaluating the extent of resection on survival.

### Univariate and multivariable cox regression analyses

For all patients, univariate analysis (UVA) demonstrated significantly improved survival for patients with younger age (HR 1.03, p < 0.001), female gender (HR 0.94, p < 0.001), black ethnicity (HR 0.89, p < 0.001), higher KPS (HR 0.51, p < 0.001), obtaining a GTR in comparison to a STR (1.29, p < 0.001), MGMT promoter-methylated gene status (HR1.51, p < 0.001), unifocal disease (HR 1.46, p < 0.001), higher RT dose (HR 0.99, p < 0.001), and a delay of RT > 4 weeks (4.1–6 weeks: HR 0.91, p < 0.001; 6.1–8 weeks: HR 0.93, p < 0.001; >8 weeks: HR 0.90, p < 0.001). On multivariable analysis (MVA), female gender (HR 0.92, p < 0.001), black ethnicity (HR 0.89, p = 0.002), higher KPS (HR 0.63, p < 0.001), higher RT dose (HR 0.99, p < 0.001), and delay of RT of 4.1–8 weeks continued to be prognostic for longer survival (4.1–6 weeks HR 0.95, p = 0.001; 6.1–8 weeks HR 0.92, p = 0.004); whereas, older age (HR 1.03, p < 0.001), obtaining a STR/biopsy in comparison to a GTR (HR 1.22, p < 0.001), MGMT promoter-MGMT promoter-unmethylated gene status (HR 1.61, p < 0.001), multifocal disease (HR 1.38, p < 0.001), and a lower RT dose (HR 0.99, p < 0.001; Table 2) were risk factors for worse survival.

**Table 2:** Univariate and Multivariate Cox Regression Analysis for Predictors of Overall Survival.

	All Patients (n = 45,942)					GTR (n = 11,470)					STR/biopsy (n = 13,594)				
	UVA HR	UVA p-value	MVA HR	MVA HR 95% CI	MVA p-value	UVA HR	UVA p-value	MVA HR	MVA HR 95% CI	MVA p-value	UVA HR	UVA p-value	MVA HR	MVA HR 95% CI	MVA p-value
Age (cont.)	1.03	<0.001	1.03	1.03–1.03	<0.001	1.03	<0.001	1.03	1.03–1.03	<0.001	1.03	<0.001	1.03	1.02–1.03	<0.001
RT Delay (weeks)															
<4	REF														
4.1–6	0.91	<0.001	0.95	0.92–0.98	0.001	0.91	<0.001	1.01	0.96–1.06	0.73	0.91	<0.001	0.90	0.86–0.95	<0.001
6.1–8	0.93	<0.001	0.92	0.87–0.97	0.004	0.93	<0.001	0.96	0.88–1.05	0.36	0.90	0.003	0.90	0.83–0.97	0.007
>8	0.90	<0.001	0.96	0.89–1.04	0.307	0.90	<0.001	1.14	1.02–1.28	0.026	0.86	0.003	0.91	0.82–1.01	0.083
Gender															
Male	REF														
Female	0.94	<0.001	0.92	0.89–0.95	<0.001	0.92	0.001	0.91	0.87–0.96	<0.001	0.94	0.005	0.92	0.88–0.97	<0.001
Ethnicity															
White	REF														
Black	0.89	<0.001	0.89	0.83–0.96	0.002	0.88	0.02	0.98	0.88–1.09	0.71	0.85	<0.001	0.83	0.75–0.91	<0.001
KPS															
<70	REF														
≥70	0.51	<0.001	0.63	0.56–0.70	<0.001	0.54	<0.001	0.64	0.53–0.77	<0.001	0.51	<0.001	0.64	0.55–0.73	<0.001
Resection															
GTR	REF														
STR/biopsy	1.29	<0.001	1.22	1.18–1.26	<0.001	—	—	—	—	—	—	—	—	—	—
MGMT promoter-Methylated															
Yes	REF														
No	1.51	<0.001	1.61	1.49–1.73	<0.001	1.74	<0.001	1.85	1.64–2.09	<0.001	1.44	<0.001	1.48	1.33–1.64	<0.001
Focality															
Unifocal	REF														
Multifocal/ Multicentric	1.46	<0.001	1.38	1.32–1.45	<0.001	1.44	<0.001	1.43	1.33–1.54	<0.001	1.41	<0.001	1.35	1.27–1.43	<0.001
RT Dose (cont.)	0.99	<0.001	0.99	1.00–1.00	<0.001	0.99	<0.001	0.99	0.99–0.99	<0.001	0.99	<0.001	0.99	0.99–0.99	<0.001

UVA: univariate analysis; HR: hazard ratio; MVA: multivariate analysis; CI: confidence interval; GTR: gross total resection; STR: subtotal resection; REF: reference variable; RT: radiotherapy; RT Delay: time from surgical resection to initiation of RT; KPS: Karnofsky Performance Status; cont.: continuous.

A total of 11,470 patients were treated with a GTR. For this cohort, UVA resulted in the following predictors for improved OS: younger age (HR 1.03, p < 0.001), female gender (HR 0.92, p = 0.001), black ethnicity (HR 0.88, p = 0.02), higher KPS (HR 0.54, p < 0.001), MGMT promoter-methylated gene status (HR 1.74, p < 0.001), unifocal disease (HR 1.44, p < 0.001), higher RT dose (HR 0.99, p < 0.001), and RT delay of >4 weeks (4.1–6 weeks: HR 0.91, p < 0.001); 6.1–8 weeks: HR 0.93, p < 0.001; >8 weeks: HR 0.90, p < 0.001). On MVA, female gender (HR 0.91, p < 0.001), higher KPS (HR 0.64, p < 0.001), and higher RT dose (HR 0.99, p < 0.001) remained significant protective factors, and older age (HR 1.03, p < 0.001), MGMT promoter-unmethylated gene status (HR 1.85, p < 0.001), and multifocal disease (HR 1.43; p < 0.001) were risk factors for poor survival. However, for patients who had a GTR, initiation of RT > 8 weeks from surgery was a risk factor for poor survival when compared to starting treatment ≤4 weeks from resection (HR 1.14, p = 0.026). There was no difference in survival for patients with an RT delay of 4.1–8 weeks when compared to <4 weeks in this cohort (4.1–6 weeks: HR 1.01, p = 0.73; 6.1–8 weeks: HR 0.96, p = 0.36; Table 2). A secondary analysis was performed to examine the significance of initiation of RT > 8 weeks from surgery using ≤8 weeks as the reference and found that a delay of >8 weeks predicted for worse survival (HR 1.23, p = 0.007).

A total of 13,594 patients underwent a STR or biopsy prior to initiation of RT. On UVA, younger age (HR 1.03, p < 0.001), female gender (HR 0.94, p = 0.005), black ethnicity (HR 0.85, p < 0.001), higher KPS (HR 0.51, p < 0.001), MGMT promoter-methylated gene status (HR 1.44, p < 0.001), unifocal disease (HR 1.41, p < 0.001), higher RT dose (HR 0.99, p < 0.001), and delay of RT > 4 weeks after surgery (4.1–6 weeks: HR 0.91, p < 0.001; 6.1–8 weeks: HR 0.90, p = 0.003; >8 weeks: HR 0.86, p = 0.003) portended an improved OS. On MVA, female gender (HR 0.92, p < 0.001), black ethnicity (HR 0.83, p < 0.001), higher KPS (HR 0.64, p < 0.001), and higher RT dose (HR 0.99, p < 0.001) remained significant positive prognostic factors of longer survival, while older age (HR 1.03, p < 0.001), MGMT promoter-unmethylated gene status (HR 1.48, p < 0.001), and multifocal disease (HR 1.35, p < 0.001) were risk factors for shorter survival. Additionally, for patients undergoing a STR or biopsy, an RT delay of 4.1–8 weeks was protective of survival when compared to initiation of RT ≤ 4 weeks from surgery (4.1–6 weeks HR 0.90, p < 0.001; 6.1–8 weeks HR 0.90, p = 0.007; Table 2).

### Survival analysis

The median OS for all patients was 14.4 months, and survival was significantly different between time interval groups. The longest survival was seen in the 4.1–6 week RT delay group with a median survival of 15.2 months, this was followed by the RT delay of >8 weeks with 14.6 months, then 6.1–8 weeks with 14.4 months, and finally, <4 weeks to RT with 13.9 months, (p < 0.0001).

## Discussion

**Figure 2:**
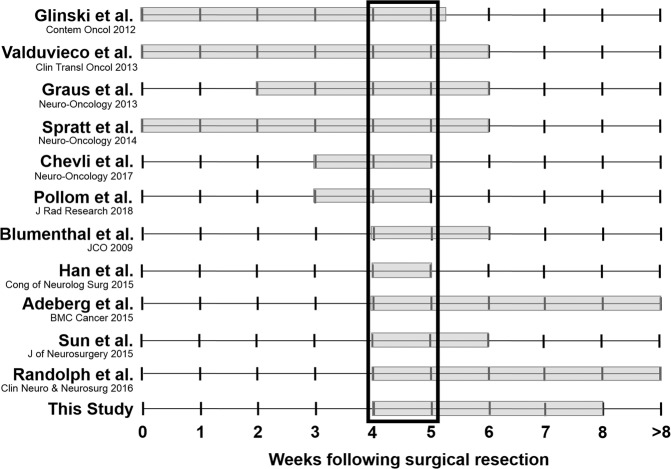
Recommended post-resection radiation delay times for Glioblastoma.

The time delay from surgical resection to the start of adjuvant RT for patients with newly diagnosed GBM is a clinically adjustable factor that can be acted upon in clinical practice and in clinical trial design. The impact of this time interval between surgery and the start of RT has been studied in a number of retrospective series and population analyses (Table 3, Fig. 2). These studies have included a few hundred to a few thousand patients treated between 1974 and 2015 with surgical resection, followed by adjuvant radiation, with or without adjuvant Temozolomide (TMZ^8–29^). Prior analyses included patients with a median RT delay ranging from 12 to 47 days and the median reported OS from these reviews ranged from 7.4 to 26 months. A total of six studies: four retrospective studies, one prospective, and one focused analysis of the NCDB only included patients treated with adjuvant radiation and TMZ^14,21,24,25,27,28^.

**Table 3:** Literature Review of Clinical Impact of Radiation Delay following Surgical Resection for Glioblastoma.

Study	Center	n	TX Dates	RT Delay (days)	Age (median)	KPS > 70 (%)	TMZ (%)	Median OS (months)	Delay of RT Conclusion
**Delay of RT is not prognostic**									
Hulshof *et al*., 2001	Netherlands, retrospective	198	1988–1998	28	—	—	0	7.4	• No difference between ≤35 or >35 days
Lutterbach *et al*., 1999	Germany, retrospective	149	1986–1997	13	60	66	0	8.8	• No difference between ≤13 or >13 days
Lopez *et al*., 2008	France, retrospective	60	2004–2006	43.5	60	100% > 60	68	14.3	• Delay not significant
Wehming *et al*., 2011	Germany, retrospective	153	2002–2008	24	58	ECOG 1	67.3	14.5	• Delay not significant
Lai *et al*., 2012	Columbia, SEER	1,375	1991–2000	15	72	—	0	GTR: 9.3, STR: 8	• Delay not significant in STR/biopsy
Noel *et al*., 2012	France, EORTC	400	2006	41	60.5	—	67	13.6	• Delay not significant
Loureiro *et al*., 2015	Brazil, retrospective	115	2003–2011	42	57	76.6	60.7	14.1	• Delay not significant
Seidlitz *et al*., 2015	Germany, retrospective	369	2001–2014	27	62	ECOG 0-2	67	18	• Delay not significant
Louvel *et al*., 2016	France, retrospective	692	2005–2011	45	57.5	65.8	100	19.7	• Delay not significant
**Delay of RT > 4 weeks worsened OS**									
Do *et al*., 2000	Australia, retrospective	182	1979–1995	26	57	ECOG 0-2	0	8.5	• Increased risk of death by 2% for each additional day of RT delay from Dx
Irwin *et al*., 2007	New Zealand, retrospective	172	1993–2003	35	59	60	0	8.5	• Increased risk of death by 8.9% for each additional week of RT delay from surgery
**Delay of RT > 4 weeks might improve OS**									
Glinski *et al*., 2012	Poland; retrospective	308	1995–2005	37	majority > 40	majority < 60	—	10% at 2 years	• Delay <5.3 weeks has better OS
Valduvieco *et al*., 2013	Spain; prospective	107	1994–2009	47	58	80	80	16.8	• GTR: Delay <6 weeks has better OS
Graus *et al*., 2013	Spain, retrospective	834	2008–2010	42	62	63.5	61	11.8	• Delay ≤6 week has better PFS
Spratt *et al*., 2014	MSKCC, prospective	345	2000–2012	31	60	88.4	100	12.2	• Delay >6 weeks worse OS than ≤2 weeks
Chevli *et al*., 2017	MDACC, retrospective	150	2007–2013	26	55	90	100	26	• Majority of patients RT delay 3–5 weeks • Not enough variance to detect signal
**Delay of RT > 4 weeks improves OS**									
Pollom *et al*., 2018	NCDB	12,738	2010–2013	29	majority 50–70	—	100	14.2	• GTR: Delay 3–5 weeks improved OS
Blumenthal *et al*., 2009	RTOG database	2,855	1974–2003	12–33	~56	76	—	12.5	• Delay 4–6 weeks has better OS (vs 2 weeks)
Han *et al*., 2015	UCSF; retrospective	198	2004–2010	29.5	~56	97	100	—	• Delay 4–5 weeks has better OS
Adeberg *et al*., 2015	Germany, retrospective	50	2004–2011	35	59	Median 90	52	16.2	• Delay to determine MGMT status did not impact survival • Delay <4 weeks has worse OS
Sun *et al*., 2015	UCSF, retrospective	218	2005–2015	27	58	—	100	15.9	• Delay >6 weeks has worse OS • Delay 4–6 weeks does not have worse PFS or OS
Randolph *et al*., 2016	Wake Forest; retrospective	161	1999–2010	27	60.8	68	71	12.2	• STR: Delay >4 weeks has better OS
This Study	NCDB	45,942	2004–2015	29	61	7*	67	14.4	• Delay 4–8 weeks has better OS • STR: Delay <4 weeks has worse OS • GTR: Delay >8 weeks has worse OS

^*^Majority of KPS scores not reported (Table 1); RT: radiotherapy; TX: treatment; KPS: Karnofsky Performance Status; TMZ: concurrent Temozolomide; OS: overall survival; GTR: gross total resection; STR: subtotal resection; chemo: chemotherapy; MDACC: MD Anderson Cancer Center; NCDB: National Cancer Database; UCSF: University of California at San Francisco; MSKCC: Memorial Sloan Kettering Cancer Center; NPS: neurologic performance scale; PFS: progression free survival.

Our large retrospective analysis of patients with newly diagnosed GBM from the National Cancer Database found that patients who have at least a 4 week interval between resection and the start of adjuvant RT have improved OS, consistent with the majority of prior published series (Tables 2–3, Fig. 2). Per our analysis, these patients experience a 1.3 month median survival benefit over patients initiated on radiation <4 weeks from surgery. Blumenthal *et al*. analyzed the RTOG database and reported findings of 2,855 patients treated from 1974–2003 with improved OS when RT was delayed 4 to 6 weeks^20^. Similarly, Han *et al*. performed a retrospective review of 198 patients treated with TMZ who were treated from 2004–2010 at the University of California-San Francisco (UCSF). They found improved OS with an RT delay of 4–5 weeks following surgery^24^. Sun *et al*., also analyzed patients from UCSF treated from 2005–2015 (n = 218) with concurrent TMZ and found that a delay >6 weeks has worse OS^28^. Randolph *et al*. reported their retrospective analysis of 161 patients from Wake Forest treated from 1999–2010 and found that when patients undergo a STR, an RT delay of >4 weeks improves survival^26^. Adeberg *et al*., retrospectively reviewed 50 patients treated between 2004–2011 in Germany and found that delaying RT to determine MGMT promotor status did not impact survival, and a delay <4 weeks had worse OS^19^. Finally, Pollom *et al*. analyzed a focused cohort of the NCDB treated with the Stupp protocol and found improved OS with an RT delay of 3 to 5 weeks for patients who undergo a GTR^25,30^.

Additionally, five studies found that a delay of less than 5–6 weeks improves survival; but they did not report significance at the 4 week cut-off point. Glinski *et al*. reported improved survival with RT delay of <5.3 weeks based on a retrospective review of 308 patients treated from 1995–2005^22^. Valduvieco *et al*. reported improved survival with a delay of less than 6 weeks following GTR in 107 patients treated from 1994–2009^29^. Graus *et al*. retrospectively reviewed 834 patients treated from 2008–2010 and found that a delay of 6 or more weeks following surgery was associated with better progression free survival (PFS^23^). Spratt *et al*. prospectively evaluated 345 patients treated with RT and TMZ at Memorial Sloan Kettering (MSKCC) from 2000–2012 and found that a delay >6 weeks had worse OS when compared to delay less than or equal to 2 weeks^27^. Finally, a retrospective review of 150 patients from MD Anderson Cancer Center by Chevli *et al*. was not able to discern a survival advantage for a specific RT delay interval; however the majority of their patients were treated 3–5 weeks following surgery, and this review reported the longest median OS of the previously published literature at 26 months^21^.

In contrast to these studies favoring an interval of several weeks between surgery and subsequent RT, two studies published in the early 2000’s reported worsened OS with any delay in RT. A retrospective analysis of 182 patients treated from 1979–1995 with an average interval between surgery and RT of 26 days reported no significant difference in outcomes associated with delay of RT following surgery or biopsy, but they found that each additional day of RT delay from initial presentation increased the risk of death by 2%^8^. In this study, Do *et al*. excluded 31 patients who had small tumors, complete resections, or were treated with radiosurgery, as well as those that received adjuvant chemotherapy. The exclusion of patients with these potentially favorable prognostic factors such as smaller tumor size and complete resections as well as adjuvant chemotherapy likely influenced the findings of this study. Irwin *et al*. published a retrospective review of 172 patients treated from 1993–2003 with an average RT delay of 35 days. In this manuscript, the authors state that each additional week of RT delay increases the risk of death by 8.9%, but this calculation was made using estimated survival curves for a hypothetical population that was derived from a proportional hazards model and not from the study population evaluated in the analysis^9^.

In our study, further analysis revealed an association between the extent of surgical resection and the impact of time between surgery and the start of RT on outcome. We found that delay of RT > 8 weeks after GTR or <4 weeks after STR was associated with worse OS, and the best survival outcomes were seen when radiotherapy was initiated 4–8 weeks following surgical resection. Specifically, in patients achieving a GTR, those initiated on RT ≤ 8 weeks from surgery had a median OS of 16.9 months, and those initiated >8 weeks had a median OS of 15.2 months (a difference of 1.7 months). In patients in whom a STR was performed, initiating RT ≤ 4 weeks from surgery resulted in a median OS of 12.9 months; whereas, starting RT > 4 weeks from surgery had a median OS of 13.8 months (a difference of 0.9 months). Prior studies that reported improved survival with an RT delay of at least 4 weeks may have had a substantial proportion of patients with a STR based on our analyses.

The mechanism of improved survival with a 4–8 week RT delay following surgery is unknown. Several hypotheses have been suggested in the literature, although no data is available as evidence of the pathophysiology. We hypothesize that some possible mechanisms might include a bias of clinicians to expedite treatment in patients who undergo a STR/biopsy or have more aggressive disease courses, rushing them to start treatment sooner. This may have contributed to the worse survival seen in patients with shorter delay between surgery and RT. In our study population, there were similar proportions of resection types within each time interval from surgery to RT. Prior studies that reported shorter survival with an interval of less than 4 weeks between surgery and RT also suggested increased complications as a result of this short time delay from surgery and the start of RT. Additionally, we have seen in our practice that when RT is initiated before 4 weeks, contrast enhancement on MRI or CT is difficult to interpret in the immediate post-operative setting. Additionally, the surgical cavity can undergo more dramatic changes within the first 4 weeks following surgical resection such that the radiation plan generated based on early post-operative imaging may not fully encompass the extent of disease throughout the course of RT^31–33^. For these reasons, allowing a delay following surgery before acquiring the RT planning imaging may facilitate better definition of the target volume and more stability of the target volume through the course of RT. Ongoing studies of serial imaging during radiotherapy may identify the impact of surgical cavity changes on RT coverage and resulting survival outcomes. Finally, one possible hypothesis to explain our finding of worsened survival when RT was delayed >8 weeks follow a GTR could be a result of re-growth of disease during this extended time delay prior to the start of RT to the point that these delayed cases of GTR represent similar tumor bulk within the radiation volume as STR cases and therefore have worse survival; although there is currently no published evidence of imaging changes following resection of GBM > 8 weeks to further evaluate this idea.

The NCDB provides a valuable tool to study large real-world cohorts and to seek answers to questions that are unlikely to be studied in a prospective randomized clinical trial. However, our study has inherent limitations due to the nature of the NCDB, which can have potential miscoding of variables, selection bias that we cannot detect including the location of the tumor in the brain, the size of the tumor, and any complications from surgery, and missing data for some variables, specifically KPS, use of TMZ, and MGMT promoter-methylation status. These three variables are likely significantly considered when treatment recommendations, including when to start radiotherapy, are being made, and it is unfortunate that more information was not provided for analysis. Secondly, the NCDB only provides all-cause survival without data on progression-free survival or cancer-specific survival.

Furthermore, numerous other prognostic factors have been found to influence survival in patients with GBM, which are not included in the NCDB database. One such factor, the influence of tumor contact with the lateral ventricles, has been shown to decrease survival in patients with GBM on a prior meta-analysis^34^. Additionally, hyperglycemia has been shown to worsen survival in GBM patients treated with surgery, RT, and TMZ^35^. Specifically, the authors found that a glucose level of >6.3 mmol/L portended for worse survival in their retrospective analysis. Finally, numerous imaging characteristics have been evaluated to determine their influence on patient survival, and the degree of necrosis and enhancement on preoperative magnetic resonance imaging studies was found to be correlative^4^.

In conclusion, this analysis of the NCDB suggests that for patients with newly diagnosed GBM, an RT delay of 4 to 8 weeks following resection is associated with better overall survival. The impact of time between surgery and RT was dependent on the extent of resection. Particularly in patients with GTR, delays of longer than 8 weeks was associated with worse survival, making them comparable to patients who receive a STR. This impact of time delay from surgery to the start of RT, in conjunction with the extent of surgery, should be considered in clinical practice and possibly in the design of future clinical trials, particularly those that require additional molecular and genetic analyses that can lead to time delays to start adjuvant treatment.
